# Studying the Hydration of a Retarded Suspension of Ground Granulated Blast-Furnace Slag after Reactivation

**DOI:** 10.3390/ma9110933

**Published:** 2016-11-18

**Authors:** Nick Schneider, Dietmar Stephan

**Affiliations:** Building Materials and Construction Chemistry, Technische Universität Berlin, Gustav-Meyer-Allee 25, 13355 Berlin, Germany; nick.schneider@tu-berlin.de

**Keywords:** retardation, reactivation, ground granulated blast-furnace slag, d-gluconic acid, sodium hydroxide, hydration study

## Abstract

This article presents a combined use of a retarder (d-gluconic acid) and an alkaline activator (sodium hydroxide) in a binder system based on ground granulated blast-furnace slag. The properties of the retarder are extending the dormant hydration period and suppressing the generation of strength-giving phases. Different retarder concentrations between 0.25 and 1.00 wt.% regulate the intensity and the period of the retardation and also the characteristics of the strength development. The activator concentration of 30 and 50 wt.% regulates the overcoming of the dormant period and thereby the solution of the slag and hence the formation of the hydration products. The research objective is to produce a mineral binder system based on two separate liquid components. The highest concentration of retarder and activator generates the highest compressive strength and mass of hydration products—after 90 days of hydration a compressive strength of more than 50 N/mm^2^. The main phases are calcium silicate hydrate and hydrotalcite. Generally, the combination of retarder and activator shows a high potential in the performance increase of the hydration process.

## 1. Introduction

The present research work examines the state of knowledge of retardation and activation of binders, after an introduction to the practical use of the binder system. These topics are often reviewed separately in other research studies (cf. [1,2,3,4,5,6,7,8,9]), thus creating the background for studying the combined use of a retarder and an activator in one binder system based on effective retardation. This work is a unique study of the retardation of a pure suspension of ground granulated blast-furnace slag (GGBFS) without any Ordinary Portland Cement (OPC) (no blended cement). The literature in the field of the retardation is mostly on pure OPC binders (cf. [7,8,10,11,12,13,14,15,16,17,18,19,20]); there are a few on blended systems of OPC plus GGBFS (cf. [21,22]), but none on pure GGBFS. However, some of the analyzed effects are the same in both binder systems (cf. [23,24,25,26]). Therefore, the literature review also describes the effects of additives that retard an OPC binder.

Based on the hydration control, individual reactivation of the retarded binder can be achieved by the addition of an activator (cf. [27,28]). The concept of the combination is based on the recycling process of fresh concrete. The addition of retarder in unused concrete interrupts the setting for some hours up to the addition of fresh concrete to reactivate the hydration. This approach will be transferred to a less reactive binder, GGBFS, which can be activated by an alkaline solution. The aim is to control the hydration process and performance increase by the combined use of a retarder and activator. A second aim is to use this binder system as a two-component system, consisting only of liquid components, for simple and quick application. Thereby, it is possible to use this system at any time just by mixing it with a static mixer. Similar multi-component systems, with almost the same idea but another binder, were also studied by Pang et al. [29] and Brothers et al. [30].

The hydration process of the OPC is based on five periods that can be varied by the application of a retarder like various types of sugar or, in particular, carboxylic acid such as d-gluconic acid [17,18,20,21,23,31,32,33,34,35]. According to the type and concentration of the retarder, the dormant period can take several times longer than the primary duration in comparison to the state without retarder [10,20,23,36,37,38]. The exact operating principles are still unclear; however, these principles are limited to the complexation of the retarder with the calcium that is involved in all hydration products or to the adsorption of the retarder of the hydrated or not hydrated surfaces of the silicate or aluminate components/products [8,9,14,19,23,39,40]. The characteristics of the retarder effect are easier to explain. At the beginning of the hydration process, the retarder prevents the formation of ettringite and if the concentration is high enough the appropriate retarder also prevents the formation of calcium silicate hydrate (C-S-H) phases [7,17,18]. Therefore, it results in a simple retardation of the setting and/or a retardation of the formation/growth of the strength-giving phases [7,8,36]. The effect of the retarder does not only affect OPC; it can also be transferred to GGBFS [23,27,34], due to the very similar hydration products when reacting with water [34,41,42,43].

The concentration of the used retarder regulates both the setting and the strength development. Therefore, an increase in strength during the advanced hydration stage is not only brought about by inorganic zinc oxide [44] but also by various plasticizers [45] and types of sugar [22,46]. The strength increase also partly arises from the reduction of the water content, which can be reduced because of the better workability due to the addition of the retarder [11,45]. An increase in strength is also possible using carboxyl acids like citric acid [13]. The research of Juenger et al. [8] provides possible reasons for the strength increase with sugar as a retarder. They showed an increased surface area and a decreased porosity in the advanced hydration stage due to the application of sugar [8]. However, retarders also have disadvantages, such as early setting after an overdose [11,15,16,22] or a general decrease in the early strength and a total failure of hardening [12,15,16,27]. Generally, the strength is strongly dependent on the retarder concentration. Therefore, a high concentration increases the advanced strength and decreases the early strength; it has the same performance for low retarder concentrations [11,13,22,28].

However, the retarder is not the only additive that has an influence on the hydration and the strength development. The alkaline activator for the activation of GGBFS also plays an important role. The main factors of the alkaline activator are the type and the concentration [3,4,28,47,48,49,50,51,52,53,54]. So, the hydration process is controllable by the specific use and the suitable content [25,28,55]. Thereby, the prior-ranking factor is the pH value, which controls the dissolution of the vitreous form of the slag, whereby the formation of C-S-H phases is initiated [4,24,48,50,53,56,57,58,59,60,61]. Sodium hydroxide (NaOH) has faster dissolution in comparison to other activators such as water glass [62,63] but a slower effect on the formation of phases [24,55]. The studies with water glass for the activation of GGBFS from Ben Haha et al. [62,63] The studies show a result between low and no strength until the first day of the early stage of hydration. However, it is known from other research studies [48,49,54,62,63,64] that water glass can generate strength-giving phases very fast and with high performance, but not in the early period of the hydration. Thus, the activator concentration and also the pH value have an effect on the whole hydration process, from the beginning of dissolution of the slag through the setting and up to the strength development.

The hydration products of the activation from GGBFS by NaOH are mainly crystalline C-S-H phases [5,53,56,65], hydrotalcite from the available Mg and Al of the slag [59,66,67] and calcite [1,60,64]. Further probable hydration products are mostly amorphous C-(A)-S-H phases, in which the Si is partly substituted by Al depending on the present amount of the elements [26,51,63,67]. Additionally, aluminate-ferrite-mono(sulphate) hydrate phases (AFm phases) were also detected via thermogravimetry by Gruskovnjak et al. [67] and Wang et al. [65]. Similar hydration products are present when calcium hydroxide is used as an activator [68,69]. However, the C-S-H phases can also be more complex due to the use of an activator like water glass or specific Ca/Si ratios in which the phases are variably extendable around the element(s) Al and/or Na [1,56,59,60,70,71,72]. The C-S-H phases of alkali-activated GGBFS are comparable to the structure of tobermorite, the modification of which is probably changeable over the hydration time [1,62,63,73,74]; the crystallinity increases over time [5,60]. According to Richardson’s model of C-S-H phases, tobermorite and jennite with a high Ca/Si ratio are possible phases in the hydration of OPC with, for example, GGBFS [74,75]. Also, Richartz [76] and Nonat [73] reported on the possible difference of the Ca/Si ratios on the basis of tobermorite. However, the formation of the phases seems to be largely independent of the type of activator used [5,62,65], which represents a contrast to the strength development. Roy et al. [5] and Song et al. [6,61] consider the pH value of the activator as the main influence factor. Song et al. [6,61] relate this influence to the different dissolution of ions, in which the behavior of Si and Ca is contrary to each other.

The aim of the present work was to study the hydration of a retarded suspension of GGBFS after reactivation. The following results show the lasting effect on the hydration and the formation of products due to the different concentrations of the used retarder. Also, the influence of the activator concentration after the reactivation of the retarded binder suspension is illustrated. The results are evaluated by the analyses of compressive strength, thermogravimetry (TG), X-ray diffraction (XRD) and scanning electron microscopy (SEM).

## 2. Materials and Methods

**Figure 1:**
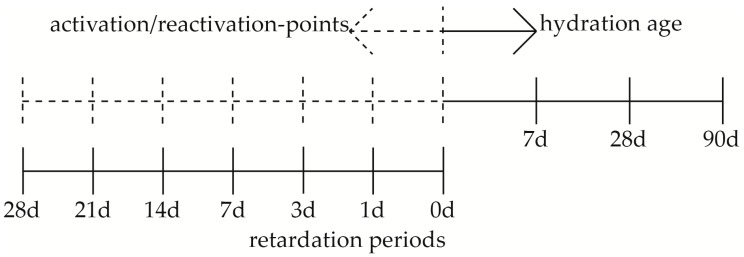
Timescale of the retardation periods (0–28 days), the points of activation/reactivation (Ac1, Ac2) and the ages of the hydration (7–90 days).

The retarded binder system consisted of GGBFS, a solution of d-gluconic acid, and methyl cellulose (MC) with a water–binder ratio (w/b ratio) of 0.30 (Table 1). GGBFS is used due to the lower reactivity and the stable retardation over a long period of time in comparison to OPC [24]. However, GGBFS has hydraulic components, and therefore is clearly differentiated from pozzolanic binders such as fly ash [41,43]. The retardation is achieved by d-gluconic acid with concentrations of 0.25, 0.50, and 1.00 wt.% with respect to the water content, while the periods of retardation are between 0 and 28 days (Figure 1). At the end of each retardation period, reactivation is carried out with NaOH (Figure 1) in two concentrations of 30 or 50 wt.% (Ac1 or Ac2), by which the w/b ratios of the systems are increased to 0.40 and 0.37, respectively (Table 1). The amount of the activator is 9.9 wt.% of the whole reactivated mass. The MC with a concentration of 0.1 wt.% with respect to the whole retarded mass is used to stabilize the retarded binder system over the long period of storage by the formation of hydrogen bonds so that no separation of water occurs (cf. [77]). For the evaluation of the retarded systems, reference systems were produced with w/b ratios of 0.40 and 0.37 and with the components of GGBFS, MC and the respective solution of the activator.

The chemical and physical properties of GGBFS are shown in Table 2. Hence, the basicity (CaO/SiO_2_) is 1.21, which is comparable to the average of German GGBFS (cf. [78]). The d-gluconic acid (from Alfa Aesar GmbH & Co. KG, Karlsruhe, Germany) has a pH value of 1.82 and a density of 1.24 kg/dm^3^, and the original solution has a concentration of 50 wt.%. The MC (from Dow Wolff Cellulosics GmbH, Bomlitz, Germany) has a density of 1.39 kg/dm^3^ and a maximum adjustable viscosity of 24,600 mPa·s. The activating solutions were produced with demineralized water and NaOH pellets (purity of over 99 wt.%). Due to the high concentrations of NaOH, pH values of over 14 resulted.

The retarded mixtures were produced with a mortar mixer (type 205 from TESTING Bluhm & Feuerherdt GmbH, Berlin, Germany) at a speed of 140 rpm. Every mixture consists of four mixing sections, each of one minute duration: the homogenization of drying agents (GGBFS and MC), the mixing with the retarder solution, a break, and a repeated mixing. Each retarded mixture had a volume of 375 cm^3^, which was stored in a hermetically sealed container (volume of 500 cm^3^) at 20 °C until reactivation occurred. For the reactivation, the retarded mass was mixed with the appropriate mass of activator once more for one minute in the mortar mixer. Thereby, the onset of the hydration occurs after the addition of the activator. The investigated hydration periods of seven, 28 and 90 days started at this point in time (Figure 1). The differently continual periods or points in time (Figure 1) of retardation, reactivation and hydration produce a nesting of various binder states.

### 2.1. Compressive Strength

EN 196-1 [80] was the basis for the sample production for the strength test. Deviant from the standard and with the aim of practical representation, smaller samples (2 × 2 × 2 cm^3^), other periods of demolding (3/7 days), another method of storage (20 °C and 100% relative humidity) and no compaction were used. After the hydration periods of seven, 28 and 90 days, six samples were tested for strength with a compression testing machine (type 2060 from Toni Technik Baustoffprüfsysteme GmbH, Berlin, Germany).

### 2.2. Thermogravimetry (TG), X-ray Diffraction (XRD), and Scanning Electron Microscopy (SEM)

The remaining samples of the compressive strength test were used for TG, XRD and SEM analyses. For preparation, the material was dried in a vacuum freeze-drying device (Alpha 1-4 LSC from Martin Christ Gefriertrocknungsanlagen GmbH, Osterode, Germany), at a pressure of approx. 1 mbar. The dried material for the analyses of TG and XRD was ground in a vibration disc mill to approx. 6900 cm^2^/g according to Blaine. The quantification of the hydration products was carried out by the measurement of TG (TG 209 F3 Tarsus from NETZSCH-Gerätebau GmbH, Selb, Germany) in the range from ambient temperature up to 1000 °C (10 K/min with a purging rate of 20 mL/min of N_2_). This means that the specific mass losses were characterized by the derivation of the TG curve (DTG) (cf. [28]). Thereby, the linear losses such as C-S-H phases are separated as far as possible from the specific mass losses. The XRD analysis was carried out by a device (type Empyrean from PANalytical GmbH, Almelo, The Netherlands) with a Cu anode and wavelength Kα, at 40 kV, 40 mA, and ambient temperature. The samples were scanned between 5° and 65° 2θ with a step size of 0.013° every 1.5 s. The measurement occurred with an automatic divergence aperture on a rotating sample table.

The SEM samples were prepared using conductive silver and sputtered by gold prior to the measurement. The pictures were taken at 20 kV with a SE detector and a pressure of approx. 1 × 10^−6^ mbar at a device (type S-4000 from Hitachi Ltd., Tokyo, Japan) with a cold field emission cathode.

The XRD and SEM analysis was carried out after hydration periods of seven and 28 days that of the TG analysis also after 90 days.

## 3. Results and Discussion

The results demonstrate a controllable reactivation with regard to the different concentrations of the retarder. Also, the results show a general performance increase in compressive strength and the mass of hydration products by the combined use of retarder and activator in one binder system.

### 3.1. Compressive Strength

**Figure 2:**
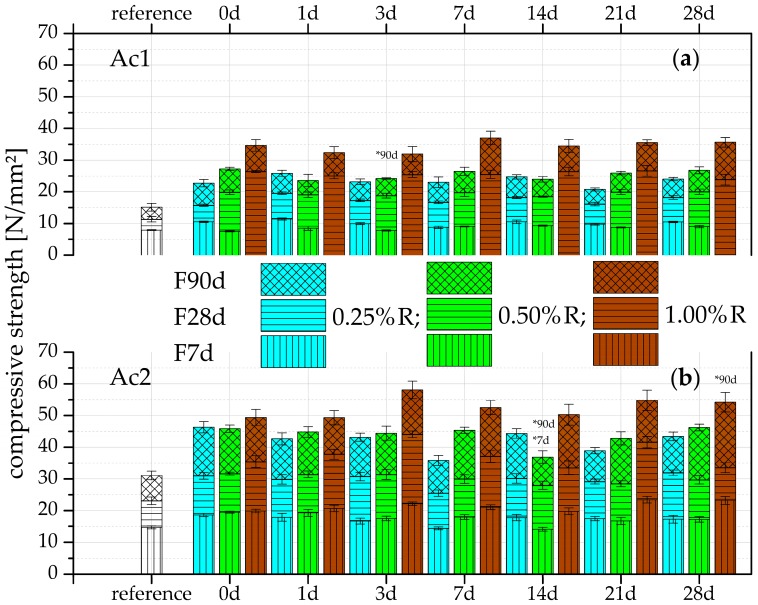
Compressive strength after seven, 28 and 90 days of hydration with (**a**) Ac1 and (**b**) Ac2 of the references (without retarder) and the reactivated mixtures with NaOH after seven different periods of retardation of 0.25%, 0.50%, and 1.00%R (* average out of four instead of six samples).

The results of the compressive strength after the different hydration periods (Figure 2) can mainly be distinguished with respect to the concentration of the used retarder and activator. There is the general correlation that with a high retarder concentration a high final strength results, but not the highest early strength (cf. [12,13,28]). Another correlation is that with the highest activator concentration the highest compressive strength is reached (cf. [28,54]).

However, these effects - the early strength and the overcoming of the retarded state after the reactivation - must be differentiated. The samples with lower retarder concentration have (Figure 2a) a higher strength after seven days of hydration. At the highest retarder concentration (1.00%R with Ac1), the formation of the strength-giving phases is prevented, hence a bonding between the slag grains does not exist and no setting occurs, which is similar to the results of Ma et al. [13]. Nevertheless, this state can be adjusted by an increase of the activator concentration, as shown in Figure 2b. This means that the overcoming of the retarded state can be accelerated by an increased activator concentration. For the fastest possible setting, the conclusion is that a low retarder concentration should be applied if a low activator concentration is present.

The results of the reference samples without retarder also show that an effective dissolution process and higher compressive strength occur with a higher activator concentration. This relation can be explained by the nature and the alkalinity of the activator. Different research studies [6,53,55] have shown a quick dissolution process and a high energy release by NaOH. These advantages were used for this binder system specifically, due to the overcoming of the probable complexation of calcium and retarder at low pH values in the dormant period. The employed high alkaline solution starts the dissolution of SiO_2_ from the slag and generates the setting and formation of C-S-H phases as fast as possible. Studies with a high alkaline sodium silicate water glass (Na_2_O/SiO_2_ approx. 16.9/27.5 wt.%) had similar qualities in overcoming the retarded state but only at a retarder concentration of 0.25%R.

The results in Figure 2 show a significant influence of the retarder by the higher compressive strength in comparison to the references. The higher strength of mixtures with retarder could be explained by the increasing surface area, which is the result of more hydration products (cf. [8]). Mixtures of GGBFS and a low retarder solution (0.05%R) have confirmed the increasing surface areas by the addition of retarders, compared to a mixture without retarder and according to the results of Juenger et al. [8]. The increase of the surface area also depends on the hydration period: the longer the hydration period, the higher the surface area. The sample without retarder had a BET surface area of 4.8 m^2^/g and the one with retarder had a surface area of 5.0 m^2^/g after 14 days of hydration (the same sample preparation as the TG analyses). Therefore, the influence of the retarder is only visible after an advanced hydration, which is indicated by the very low surface area of the retarded sample after one day of hydration (with retarder 1.1 m^2^/g and without retarder 2.0 m^2^/g).

The uniform results of every retarder concentration over the retardation periods in Figure 2 show that the duration of the retardation or the dormant period has a minor effect on the compressive strength. This supports the statement that the main factors are the concentration of the retarder and activator. The same dependences are indicated after the inspection of the relative growth rates. However, the relative growth rates also show that the rates are increasing with the rise of the retarder concentration and that the rates of both activators with the lowest retarder concentration (0.25%R) are nearly the same. It can be concluded that the influence of the retarder is significant only with concentrations higher than 0.25%R, while the concentration of the activator is more important for strength development. This difference is also evidenced by the comparison of the reference samples of Ac1 with 15.1 N/mm^2^ and Ac2 with 30.1 N/mm^2^ after a hydration period of 90 days. The disparity of the w/b ratios of 0.03 can be neglected. Further investigations with GGBFS and water with a difference in the w/b ratio of 0.05 (0.30 and 0.35) show nearly the same compressive strength for all investigated hydration periods (seven, 28 and 90 days). For example, the compressive strength on the 90 days of hydration was 41.9 N/mm^2^ for the system with a w/b ratio of 0.35 and 39.8 N/mm^2^ for a ratio of 0.30 with a standard deviation of 2.5 and 0.8 N/mm^2^, respectively.

### 3.2. Thermogravimetry (TG)

**Figure 3:**
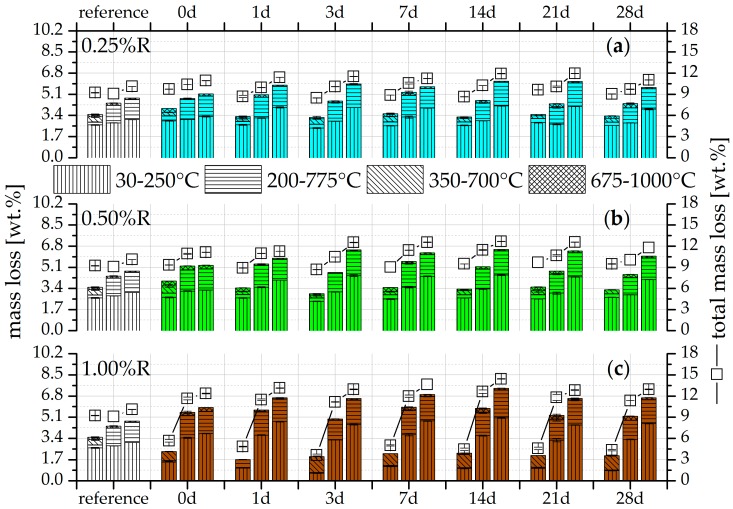
Mass loss by TG of Ac1 after seven, 28 and 90 days (from left to right, per test) after the beginning of the hydration of the reference of Ac1 (without retarder) and the reactivated mixtures with NaOH after seven different periods of retardation with (**a**) 0.25%; (**b**) 0.50%; and (**c**) 1.00%R.

**Figure 4:**
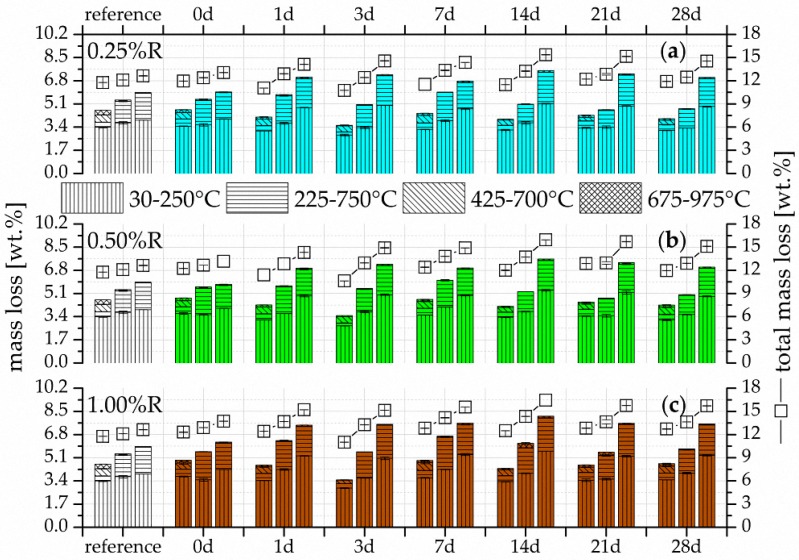
Mass loss by TG of Ac2 after seven, 28 and 90 days (from left to right per test) after the beginning of the hydration of the reference of Ac2 (without retarder) and the reactivated mixtures with NaOH after seven different periods of retardation with (**a**) 0.25%; (**b**) 0.50%; and (**c**) 1.00%R.

The TG results of the mass losses of the different temperature ranges and total mass losses for the three retarder concentrations are given in Figure 3 and Figure 4 for the two activator concentrations of Ac1 and Ac2, respectively. In addition, the figures show the results of the references from Ac1 and Ac2 and the seven different durations of retardation after the hydration periods of seven, 28 and 90 days.

More hydration products are formed by the longer hydration process and also the increases in concentration of the retarder and activator. The results also show that the reactivations yield the same or more hydration products at the latest after 28 days of hydration in comparison to the reference without retarder. The reactivations of Ac2 (Figure 4) already surpass the references at seven day of hydration. This demonstrates the positive effect of the retarder on the hydration process but also the more effective activation by Ac2. The samples of Ac1 with a retarder concentration of 1.00%R have the lowest increase of hydration products after seven days of hydration (Figure 3c), which correlates with the results of the compressive strength (Figure 2a). The remaining TG results correlate with the compressive strength as well. Generally, this analysis method demonstrates the potential of the performance increase by the rising hydration products due to the addition of the retarder.

The variance of the single reactivations is very low, similar to the results of the compressive strength. A deviation exists preferentially for short-time retardations (0 day). These reactivations show, mostly after seven days of hydration, an increased phase generation but no high growth rates after this time, in comparison to samples with a longer period of retardation. It can be assumed that the adsorption of the retarder on the binder grains is the main factor. Potentially, the retarder does not have enough time to develop its whole effect if the retarder and activator are added at nearly the same time. The influence of the retarder in binder systems—the generation of a finer microstructure and a higher surface area—is mostly not present sooner than the advanced hydration state (cf. [8]). The samples with reactivation after 0 day of retardation have a lower increase in the total mass losses or hydration products compared to those after a longer retardation time after a hydration period of 90 days (Figure 3 and Figure 4). Furthermore, more hydration products are generated with an increase in the concentration of retarder and activator. The dissolution of the slag can possibly occur almost simultaneously with the addition of retarder and activator because probably few or even no complexations of calcium and retarder must be overcome. The consequence of a fast dissolution is an increase in the alkalis in the solution (cf. [6,53,55]), which is likely to generates a dynamic hydration process, a rising pH value, and also a limited retarded effect.

The main factor influencing the formation of hydration products of the single temperature ranges is the duration of hydration. Nevertheless, a difference exists in the mass losses between the activator or the activator concentration and the retarder concentration. Thus the samples, which are reactivated from 1.00%R retardation by Ac1 after 7 days of hydration (Figure 3c), have only a low amount of C-S-H phases. These phases can be assigned mainly to the temperature range from 30 to 250 °C (cf. [5,81]). In addition, this range can also be assigned to the low probable present AFm phases, which were already detected by Wang et al. [65] and Gruskovnjak et al. [67]. However, the occurrence of these phases is rather irregular, for which the explanation is complicated. In the temperature range between 350/425 to 700 °C after seven days of hydration, a uniform intermediate product arises in all samples including the references, the development of which is independent of the retarder and activator. However, the results after seven days of hydration in Figure 3c show that the amount of this product depends on the degree of hydration. At 28 days of hydration, the intermediate products convert in the temperature range from 200/225 to 750/775 °C, which can be assigned to the magnesium-containing phases and particularly to hydrotalcite (cf. [62,82]). The research studies from Palmer et al. [82] and Cavani et al. [83] represent the different composition and also the losses of hydrotalcite at different temperature ranges. According to Palmer et al. [82], one influence factor is, for example, the alkalinity of the formation environment and, according to Cavani et al. [83], the present elements for the generation of hydrotalcite. Possibly, these and the alkalinity are the main reasons for the wide temperature range of hydrotalcite. The temperature range in which hydrotalcite is detectable has a substantial increase of hydration products in every sample from seven days to 28 days of hydration. Song et al. [61] already described the dependency of the hydrotalcite development on the hydration degree. They analyzed the phases not sooner than after 28 days of hydration with the XRD. In the research presented here, the amount of these phases depends on the degree of hydration because they already exist after seven days of hydration. The low mass losses in the temperature range from 675 to 975/1000 °C can be assigned to the carbonates (cf. [5]).

### 3.3. X-ray Diffraction (XRD)

**Figure 5:**
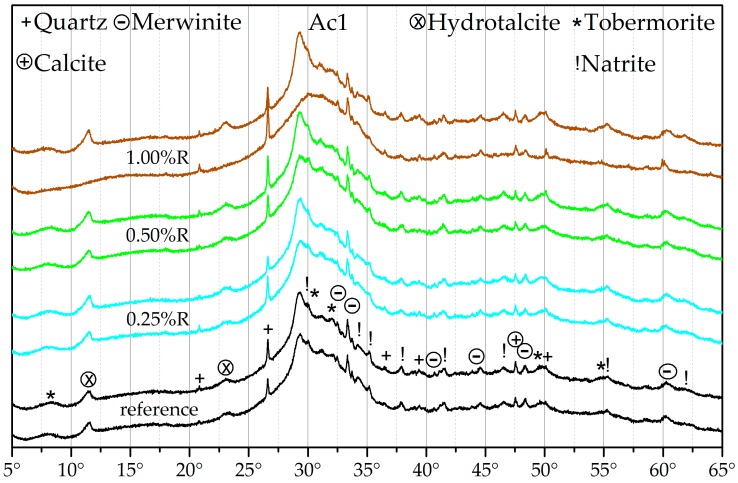
X-ray diffractogram of the reference of Ac1 (without retarder) and the reactivated mixtures with Ac1 after seven days of hydration of 0.25%, 0.50%, and 1.00%R—after hydration periods of seven (lower curve) and 28 days (upper curve) per test, with an angular range of 2θ.

**Figure 6:**
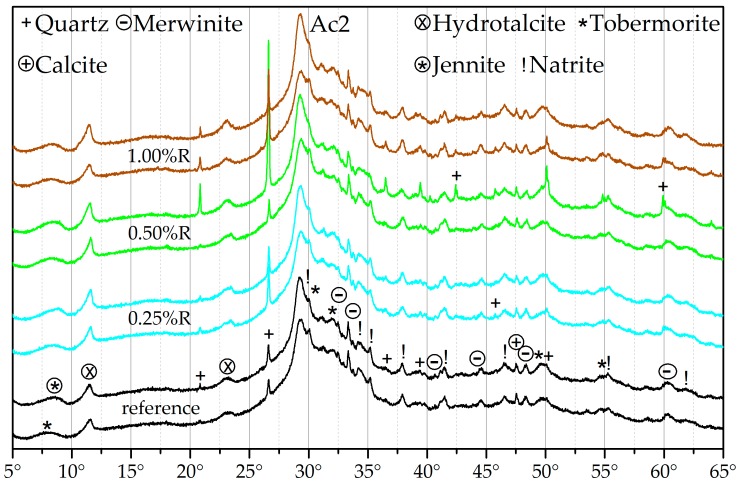
X-ray diffractogram of the reference of Ac2 (without retarder) and the reactivated mixtures with Ac2 after seven days of retardation of 0.25%, 0.50%, and 1.00%R—after hydration periods of seven (lower curve) and 28 days (upper curve) per test, with an angular range of 2θ.

Figure 5 and Figure 6 show diffractograms of Ac1 and Ac2 for the hydration periods of seven and 28 days. The reactivations of the three different retarder concentrations had a retardation period of seven days before activation. The represented results are typical examples for all periods of retardation.

GGBFS as raw material, which is not shown in the graphics, has a large amount of amorphous phases in the angular range from 22° to 37° 2θ and the few crystalline phases are merwinite, quartz, and calcite (cf. [84]). A hydration of GGBFS with demineralized water shows only a very low transformation of these amorphous phases up to 28 days of hydration. Therefore, more crystalline phases than those of the raw material are not readily detectable. In contrast to the activation with water, the samples with the alkali activator (references) show an obvious decrease in amorphous phases and an increase in crystalline phases, in particular the C-S-H phases, hydrotalcite, and natrite. However, the natrite phases are probably the result of the sample preparation and the resulting carbonation of the remnants from the activator and therefore no hydration product (cf. [85]). Another possible reason is a reaction between the retarder and the activator. Nevertheless, the natrite phases also exist in the references. Generally, the present natrite phases show an oversaturation of the activator in the mixture, which, however, was necessary for the overcoming of the retarded state and the prompt reactivation of the mixture. TG analyses of ground pure NaOH illustrate the mass loss of dehydration at approx. 300 °C and for decarbonation from 700 °C. The mass losses were increased at a longer hold-up time until the beginning of analysis due to the hygroscopic property of NaOH and the generation of carbonate like natrite. The results of the systems with and without retarder (Figure 5 and Figure 6) illustrate no obvious differences in the hydration products. However, the systems with Ac2, in contrast to those with Ac1, have a qualitative advance in the hydration degree, which can be characterized by the obviously lower amount of amorphous phases and a larger amount of crystalline phases at every time of the hydration.

The results of the analyses of TG and XRD correlate qualitatively. With both methods, it is possible to characterize the hydration products of C-S-H phases, hydrotalcite, and the carbonates that are probably represented by the natrite. The increase of crystalline phases in combination with the decrease of the amorphous phases is visible particularly in both hydration states of the reactivation with Ac1 after a retardation of 1.00%R (Figure 5). After seven days of hydration, only the natrite phases are detectable, in addition to the crystalline phases of the raw material. The other phases, which were detectable by the TG analysis, are presumably covered by the amorphous hump. At 28 days of hydration, the amorphous hump is decreased and the crystalline hydration products are recognizable. Also, the crystallinity is visible at 28 days of hydration by 0.50%R with Ac2 in Figure 6. This shows that the detection of crystalline products in this binder system depends on the hydration degree and also on the decrease of the amorphous hump (cf. [61,84]). Therefore, a characterization of the C-S-H phases in the angular range from 28° to 32° 2θ, which are hidden by the hump and underdeveloped by the effect of the retarder, is not really possible at the beginning of the hydration (cf. [86]). The main factor influencing the increased formation of phases according to Figure 5 and Figure 6 is the activator and its concentration. After the overcoming of the retarded state, the growth of the hydration products is faster at a higher activator concentration, whereby more amorphous structure can be dissolved.

The structure of the generated C-S-H phases can be assigned to that of tobermorite or jennite (Figure 5 and Figure 6). In addition, the results of the reactivation by Ac2 and the ongoing hydration process show a transformation in the C-S-H phases and therefore in the Ca/Si ratio. Richardson [74,75] has described the transformation by the structural model to the formation of C-S-H phases in mixtures with OPC. However, Richardson assumes that the alkali activation of GGBFS generates only tobermorite as C-S-H phases and no additional phases like jennite [74]. The difference of the Ca/Si ratio in the OPC phases with a variation of the Ca content was also described by Richartz et al. [76]. In addition, Nonat [73] characterizes the generally variable texture of C-S-H phases and the similarity to the phases of tobermorite and jennite according to the findings of Taylor and Richardson. The research of the activation of GGBFS from Song et al. [6,61] shows the dependence of the hydration products on the pH value of the binder system because the dissolving power of Si is very low at a pH value of 11.5. This lead to the assumption that the dissolving of Si occurs preferably at the beginning of the hydration due to the high activator concentrations in which lower Ca/Si-rates and also tobermorite-similar phases are generated. The pH value most likely drops in both activator systems during the hydration process, by which the dissolving of the Si also decreases and that of Ca increases (cf. [61]). By the assumed decrease of the Si dissolving, the systems with Ac2 have more intensive dissolving of Ca, whereby the Ca/Si rate rises and jennite-like phases originate.

The reflection range from 29° to 29.5° 2θ can be assigned to calcite and also C-S-H phases (cf. [85,86]). The high intensity of the reflections illustrates the effect of the two activator concentrations. There could be a correlation between the different compressive strength of the systems with Ac1 and Ac2 (Figure 2) and the reflection intensity or crystallinity. A comparison of Figure 2 with Figure 5 and Figure 6 shows a higher compressive strength at a higher crystallinity. The XRD analyses gave no indications for crystalline AFm phases (cf. [65,67]), brucite, or calcium hydroxide (cf. [5]). Further C-S-H phases with a modification of Na and/or Al (cf. [1,62,81]) were also not identified, most likely due to the X-ray amorphous structure of these phases.

The hydrotalcite phases increased from seven to 28 days of the hydration, which can be seen both in the results of the TG (Figure 3 and Figure 4) and of the XRD (Figure 5 and Figure 6). This increase can be realized very well at the reflection of approx. 11.5° 2θ in Figure 5 with a retarder concentration of 1.00%R. In addition, the reflection at approx. 23.0° 2θ also confirms the increase of hydrotalcite (cf. [65,87,88,89]). In this range, the reflection peak takes a narrower shape in the ongoing hydration process (Figure 6 with 1.00%R). The general broadness of the reflection at approx. 23.0° 2θ is also generated by phases of calcite and merwinite. These results confirm the findings of Song et al. [61], who state that the formation of hydrotalcite depends on the hydration degree. Nevertheless, the results show that the formation of hydrotalcite is possible within seven days of hydration with a low retarder and a high activator concentration. This quick formation is promoted by the dissolving power of the high activator concentration. The dependence of the hydrotalcite formation on the activator concentration was also described by Burciaga-Díaz et al. [1]. A further influence factor of the hydrotalcite formation, the composition of the GGBFS with its amount of Mg and Al [1,62], is not variable in the research presented here. Ben Haha et al. [62] assume that low amounts of these elements rather generate strätlingite and high amounts of brucite. However, both phases were not detectable. The existence of phases similar to zeolite was not verifiable either (cf. [90]). The pre-called phases were not detected, perhaps because of the variable composition of hydrotalcite, which is described by Palmer et al. [82], Cavani et al. [83], and Rozov et al. [88], and the constant binder composition.

The detected hydration products are normal for the alkali activation of GGBFS, which complies with a number of research studies (cf. [1,2,61,65,74,81]). Due to the results of the XRD analyses, it can be assumed that the combined use of retarder and activator in one binder system has no influence on the type of the generated phases.

### 3.4. Scanning Electron Microscopy (SEM)

**Figure 7:**
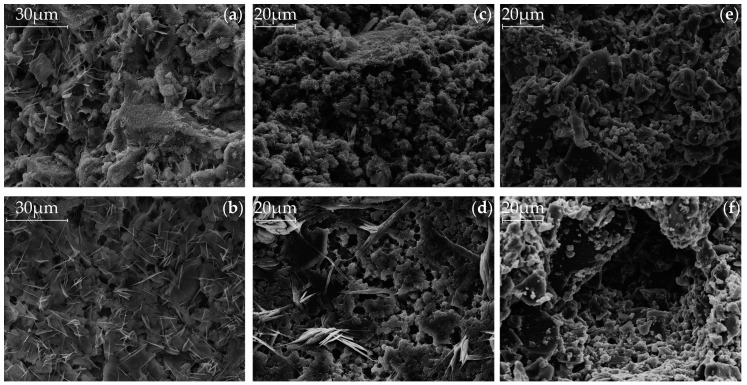
SEM pictures after a hydration period of seven days, of the reference ((**a**) of surface and (**b**) of pore) and the reactivated mixtures with Ac1 after seven days of retardation ((**c**) of surface and (**d**) of pore with 0.25%R, (**e**) of surface and (**f**) of pore with 1.00%R).

**Figure 8:**
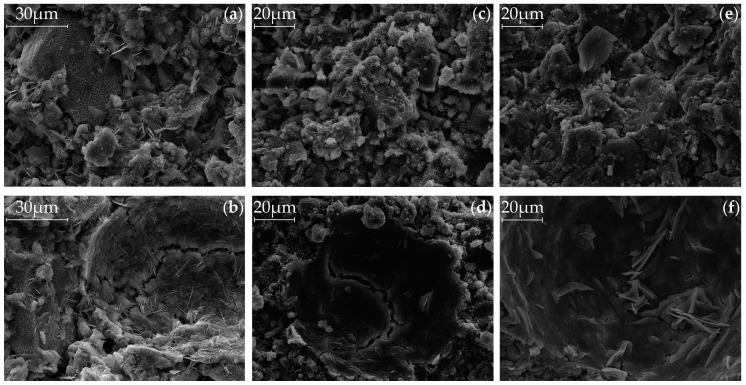
SEM pictures after a hydration period of seven days, of the reference ((**a**) of surface and (**b**) of pore) and the reactivated mixtures with Ac2 after seven days of retardation ((**c**) of surface and (**d**) of pore with 0.25%R, (**e**) of surface and (**f**) of pore with 1.00%R).

The previous results can be supported by the SEM pictures of Figure 7 and Figure 8, which show the samples after the use of Ac1 and Ac2, respectively, with the same magnification after a hydration period of seven days. Both figures are represented by (a) and (b) for the reference sample without retarder, (c) and (d) for the reactivation after a retardation of seven days and 0.25%R, and (e) and (f) for the reactivation after a retardation of seven days and 1.00%R, respectively. Figure 7 and Figure 8 can be distinguished by the pictures of the surface ((a), (c) and (e)) and those of the pore ((b), (d) and (f)).

By comparing the microstructure of the systems with Ac1 (Figure 7) and Ac2 (Figure 8), the porosity and hence the hydration degree can be differentiated. Samples with Ac2 show a denser microstructure for the reference as well as the reactivations in contrast to these of Ac1. This property is illustrated clearly by the overgrown and closed pore walls in Figure 8b,d,f. The microstructures of systems with Ac1 have a significantly lower hydration degree at the same hydration period (Figure 7b,d,f). In addition, the phases do not have the same compactness as the Ac2 systems and the space between the slag grains is not completely filled. The difference in the hydration degree can be mainly regulated by the activator concentration (cf. [6,27,28]). To a certain extent, the decrease of the retarder concentration can also contribute to a higher hydration degree; however, this is probably because of the earlier overcoming of the retarded state (compare (c) and (e) in Figure 7). The slag grains of the reactivation with Ac1 and a retardation of 1.00%R (Figure 7e,f) show only a superficial bonding but no visible hydration products. This result correlates with the findings of the analyses of compressive strength, TG and XRD. The different results in the compressive strength (Figure 2) and the mass of hydration products (Figure 3 and Figure 4) from the two activators can be explained by the visible difference of the hydration degree (Figure 7 and Figure 8). A comparison of the reference of Ac2 (Figure 8a,b) and its reactivations (Figure 8c,f) illustrates the positive influence of the retarder with the effect of a denser microstructure of the hydration products after the overcoming of the retarded state, similar to the results of compressive strength and TG (cf. [8,28]).

At the hardened state, the microstructures of alkali-activated systems have a three-dimensional structure of C-S-H phases in the range between the slag grains (outer products), and the originated network consists of strength-giving phases grown together and into each other (cf. [75,76]). In Figure 7b,d, the foil-like morphology, which was already characterized by Richardson [74] as the outer products of alkali-activated GGBFS, can be observed. According to Richardson [74], mixtures with GGBFS generally have a fine scale and foil-like morphology at low or high Ca/Si ratios. Another visible characteristic hydration product (Figure 7a) is embedded in C-S-H phases and looks like hexagonal plates (cf. [74,87]). These phases can be assigned to the hydrotalcite due to their appearance (cf. [87]). Cavani et al. [83] describe the appearance of the natural hydrotalcite as a foliated structure with contorted plates or as a fibrous mass. The rising structures in Figure 7d, for example, are similar to this description. In contrast to the previous structure assignment, Roy et al. [5] assign calcium hydroxide or also magnesium–calcium aluminate hydrate to similar rising hexagonal crystals in Figure 7b or Figure 8b. Wang et al. [65] contradict these assignments and refer to the main hydration products of C-S-H phases and hydrotalcite from alkali-activated GGBFS. The natrite, which probably are from carbonation, might also be observable in this analysis method due to the sample preparation. However, this phase is not assignable to the hydration products of Figure 7 and Figure 8.

The very close and fine-scale hydration surface stands for a high hydration degree. This statement is supported by the comparison of the SEM pictures of the retardation of 0.25%R (Figure 7c,d) and 1.00%R (Figure 7e,f) and also of the corresponding results of the compressive strength (Figure 2a), TG (Figure 3a,c) and XRD (Figure 5). In contrast to Ac1, the microstructure of the systems with Ac2 (Figure 8) has almost the same morphology and is nearly independent of the addition of the retarder, after a hydration period of seven days. The previous results (Figure 2b, Figure 4, and Figure 6) from Ac2 show similar findings. At a later stage of hydration, the microstructures of the systems with Ac1 and Ac2 are even denser. However, a difference between the references and the reactivations becomes evident. Yet the increase of hydration products in the reactivated systems arises from the addition of a retarder, which also had a positive effect on the compressive strength or the mass of the hydration products.

The results of the analyses have shown that an earlier overcoming of the retarded state is possible by increasing the activator concentration. It was also shown that with the beginning of hardening and a progressed dissolution process more hydration products can be generated. The outcome is a denser crystalline matrix with a higher compressive strength and nearly no influence of the period of retardation. Thereby, the used retarder supports the formation of more hydration products, which in turn has a positive effect on the compressive strength, especially with increased concentrations. The hydration degree is also affected by the retarder because at a lower retarder concentration the hardening process occurs earlier. Thus, the hydration has a longer period of reaction compared to higher retarder concentrations.

## 4. Conclusions

To summarize the research, the combined use of retarder and activator with GGBFS is a complex system in which the regulation of the hydration process is possible with different retarder and activator concentrations over a period of 28 days. The effect of the performance increase is also available by use of a retarder. For this two-component system, the type, concentration and consequently the pH value of the activator are generally crucial for the overcoming of the retarded state toward the dissolving of the slag grains. The research study has presented a binder system that consists of only liquid components and can be used at every stage of retardation.

The results have illustrated that the benefits from the high activator concentration including not only the overcoming of the retarded state but also the decrease of the amorphous GGBFS amount and therefore the formation of crystalline phases. The main hydration products are C-S-H phases and hydrotalcite. It could be seen that the C-S-H phases and their Ca/Si ratio vary depending on the hydration degree and thus that of the activator concentration. The systems with a higher activator concentration could be assigned to a tobermorite-like phase for early hydration and a jennite-like phase for a long hydration of up to 28 days. All systems show an increase in the hydrotalcite from seven to 28 days of hydration, which is independent of the activator or retarder concentration. An additional constituent is natrite as a carbonaceous phase, which is probably formed due to the uptake of CO_2_ during sample preparation.

The main factors that contribute to a higher compressive strength, a higher share of crystalline hydration products and a denser microstructure are: the activator concentration in the first place, followed by the hydration period and the use or amount of retarder. The development of the phase type is independent from the use of the retarder. Also, the period of the retardation has nearly no influence on the hydration process. The use of a retarder has many positive effects, such as the suppression of the hydration process for at least 28 days with all used concentrations, but also a negative aspect, which is the increase of the setting time after the reactivation. The most effective combination of a long retardation, a fast setting after reactivation and an increase of the hydration degree is the system with concentrations of 0.25 wt.% d-gluconic acid and 50 wt.% NaOH.

## Figures and Tables

**Table 1:** Composition of the retarded and reactivated binder with the w/b ratio.

Components	Retarded Binder [wt.%]	Reactivated Binder [wt.%]	
GGBFS	76.9	69.2	
Retarder solution	23.0	20.8	
MC	0.1	0.1	
Activator solution	-/-	9.9	
Activator	-/-	Ac1	Ac2
w/b ratio	0.30	0.40	0.37

**Table 2:** (**a**) Chemical composition and (**b**) physical properties of GGBFS.

**(a)**	**[wt.%]**
SiO_2_	35.6
Al_2_O_3_	10.6
Fe_2_O_3_	0.7
MnO	0.2
MgO	7.4
CaO	43.2
Na_2_O	0.2
K_2_O	0.4
TiO_2_	0.7
**(b)**	
Density	2.92 kg/dm^3^
Specific surface area (Blaine)	3218 cm²/g
Water demand (Puntke)	19.1 wt.%
RRSB-Distribution x_0_/n ^(^*^)^	20.7/1.7

^(^*^)^ The particle distribution is expressed by the distribution of Rosin, Rammler, Sperling & Bennett (RRSB) with the local parameter (x_0_) at a pass of 63.2 vol.% and also the gradient parameter (n) [79].
